# Vaginal progesterone to reduce preterm birth among HIV-infected pregnant women in Zambia: a feasibility study protocol

**DOI:** 10.1186/s40814-017-0170-7

**Published:** 2017-07-18

**Authors:** Joan T. Price, Katie R. Mollan, Nurain M. Fuseini, Bethany L. Freeman, Helen B. Mulenga, Amanda H. Corbett, Bellington Vwalika, Jeffrey S. A. Stringer

**Affiliations:** 1 Division of Global Women’s Health, Department of Obstetrics and Gynecology, University of North Carolina at Chapel Hill, 3009 Old Clinic Building, Campus Box 7577, Chapel Hill, 27599-7577 NC USA; 2 Department of Obstetrics and Gynaecology, University Teaching Hospital, Lusaka, Zambia; 3 Center for AIDS Research, School of Medicine, University of North Carolina at Chapel Hill, Chapel Hill, NC USA; 4 Pharmaceutical Society of Zambia, Lusaka, Zambia; 5 Eshelman School of Pharmacy, University of North Carolina at Chapel Hill, Chapel Hill, NC USA

**Keywords:** Preterm birth, Progesterone, HIV, Antiretroviral therapy, Sub-Saharan Africa

## Abstract

**Background:**

Women infected with HIV have a risk of preterm birth (PTB) that is twice that among uninfected women, and treatment with antiretroviral therapy (ART) may further increase this risk. Progesterone supplementation reduces the risk of preterm delivery in women who have a shortened cervix in the midtrimester. We propose to study the feasibility of a trial of vaginal progesterone (VP) to prevent PTB among HIV-infected women receiving ART in pregnancy. Given low adherence among women self-administering vaginal study product in recent microbicide trials, we plan to investigate whether adequate adherence to VP can be achieved prior to launching a full-scale efficacy trial.

**Methods and design:**

One hundred forty HIV-infected pregnant women in Lusaka, Zambia, will be randomly allocated to daily self-administration of either VP or matched placebo, starting between 20 and 24 gestational weeks. The primary outcome will be adherence, defined as the proportion of participants who achieve at least 80% use of study product, assessed objectively with a validated dye stain assay that confirms vaginal insertion of returned single-use applicators. Secondary outcomes will be study uptake, retention, and preliminary efficacy. We will concurrently perform semi-structured interviews with participants enrolled in the study and with women who decline enrollment to assess the acceptability of VP to prevent PTB and of enrollment to a randomized controlled trial.

**Discussion:**

We hypothesize that VP could prevent PTB among women receiving ART in pregnancy. In preparation for a trial to test this hypothesis, we plan to assess whether participants will be adherent to study product and protocol.

**Trial registration:**

ClinicalTrial.gov, NCT02970552.

## Background

An estimated 15 million babies are born prior to 37 weeks of gestation every year, one million of whom die as a direct result of their prematurity [1]. Over 60% of the world’s preterm deliveries occur in Africa and South Asia; in sub-Saharan Africa, the rate of preterm birth (PTB) approaches 20%, compared to rates below 10% in the Global North [2]. This disparity can be partly explained by the elevated risk of PTB faced by HIV-infected women, some 85% of whom live in sub-Saharan Africa [3]. Expanded access to HIV antiretroviral therapy (ART) has transformed the health of mothers and can prevent most cases of vertical transmission. However, an unanticipated consequence has arisen: ART use in pregnancy appears to increase the risk of PTB even beyond that attributable to HIV infection itself [4–14].

Declining concentrations of circulating progesterone and reduced progesterone activity are associated with preterm labor [15]. With supplementation, higher serum concentrations of progesterone have incremental protection against preterm labor [16]. Prenatal progesterone—an anti-inflammatory hormone administered intramuscularly or vaginally—can reduce the risk of PTB in women who have had a prior spontaneous PTB and in those with evidence of cervical shortening via midtrimester sonography [17–20]. HIV infection causes immune activation and inflammation, both systemically and in the lower genital tract [21, 22]. While suppressive ART generally improves systemic inflammation, its inconsistent effect on the lower genital tract may underlie the observed amplification of PTB risk [23–25]. Progesterone has been tested to reduce PTB for numerous high-risk indications, but has yet to be studied in HIV-infected women.

Our objective is to determine whether VP prophylaxis may reduce PTB among HIV-infected women receiving ART in pregnancy. We introduce the study protocol for a pilot study investigating the feasibility of a randomized controlled trial (RCT) of VP among HIV-infected gravidas taking ART in Lusaka, Zambia. Given recently reported challenges to adherence among young women in vaginal microbicide and HIV pre-exposure prophylaxis (PrEP) trials in sub-Saharan Africa [26, 27], it is prudent to investigate whether adequate adherence can be achieved with VP prior to launching a full-scale efficacy trial. In this mixed method design, we plan a pilot study in which 140 participants are randomized to either VP or placebo and a qualitative component investigating the acceptability of such a study. The results of these activities will allow the iterative development of study-specific standard operating procedures meant to optimize uptake, enrollment, adherence, and retention for a full-scale efficacy trial.

## Methods and design

### Study design

**Fig. 1:**
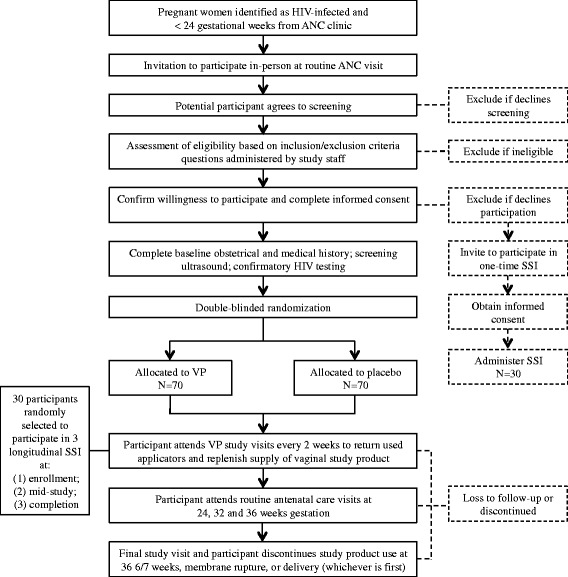
Participant flow diagram

This is a mixed method study to evaluate the feasibility and acceptability of a RCT of VP to prevent PTB among HIV-infected Zambian women. To assess the feasibility of a full-scale efficacy trial, we will implement a pilot double-blinded placebo-controlled trial of VP among HIV-infected pregnant women attending antenatal care (ANC) at the University Teaching Hospital (UTH), the referral hospital in Zambia’s capital city, Lusaka. To assess acceptability of a RCT to test VP among this population, we will employ a qualitative approach of longitudinal semi-structured interviews (SSI) among women agreeing to trial participation and a one-time SSI among those who decline to participate. This study has been designed in accordance with the Standard Protocol Items: Recommendations for Interventional Trials (SPIRIT 2013) [28] (Fig. 1, participant flow diagram).

### Study participants

Pregnant women who meet the following criteria will be eligible for enrollment: (1) 18 years of age or older; (2) viable intrauterine pregnancy confirmed by ultrasound; (3) presentation to ANC prior to 24 weeks gestation; (4) antibody-confirmed HIV-1 infection; (5) initiating or continuing ART treatment in pregnancy; (6) ability and willingness to provide written informed consent; and (7) willing to adhere to study visit schedule. We will exclude from study participation women with any of the following: (1) multiple gestation; (2) non-research indication for antenatal progesterone supplementation (i.e., prior spontaneous PTB and/or cervical length ≤20 mm on screening ultrasound); (3) planned or in situ cervical cerclage; (4) evidence of threatened abortion, preterm labor, or ruptured membranes during the current pregnancy; (5) major fetal anomaly detected on screening ultrasound; (6) known uterine anomaly; and (7) known or suspected allergy or contraindication to VP or placebo components.

### Intervention

Participants will be randomly assigned in a 1:1 ratio to intervention or placebo in a pilot RCT. The intervention group will self-administer one 200 mg VP suppository daily starting prior to 24 weeks gestational age (GA); the control group will self-administer indistinguishable placebo daily starting prior to 24 weeks GA. Participants and study staff will be masked as to treatment allocation.

The qualitative activities of this study will involve two groups. Women who decline enrollment into the randomized pilot study will be invited to participate in a one-time face-to-face SSI, while a random sample of the women choosing to participate in the RCT will be followed longitudinally with three serial SSIs.

### Primary objective

Our primary objective in this trial is to determine whether a full-scale efficacy trial will be feasible in this setting. Toward this end, we will study three key indices of feasibility: (1) study uptake, defined as the proportion of eligible women who agree to be enrolled; (2) adherence to study product, defined as the proportion of women taking ≥80% of prescribed product doses; [17, 29]; and (3) study retention, defined as the proportion of women enrolled for whom we are able to ascertain both the date of delivery and the infant vital status to define the PTB outcome. We will assess feasibility of a phase III trial with the following criteria for marking success:

1. At least 50% of eligible participants agree to be enrolled.
2. At least 70% of participants achieve adequate adherence (i.e., ≥70% of women taking ≥80% of prescribed study product doses).
3. At least 90% of participants complete the study (i.e., evaluable for PTB outcome).

We expect the feasibility study to meet all of the above criteria to satisfy requirements for progression to a phase III trial.

### Study procedures

**Fig. 2:**
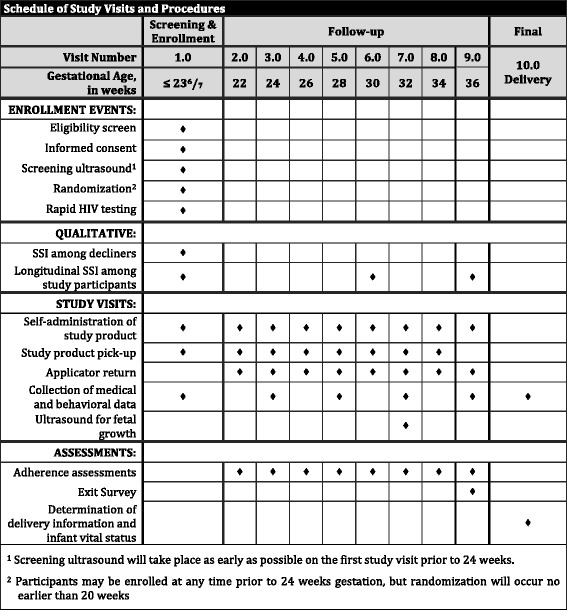
Schedule of study events, visits, and assessments

Potential participants will be identified at an early ANC visit prior to 24 weeks GA (Fig. 2). A study nurse will approach patients who may be preliminarily eligible by GA criteria based on reported last menstrual period. After an information session and determination of potential eligibility, those choosing to participate will undergo an informed consent process in their preferred language: English, Nyanja, or Bemba. Study staff will verify antenatal and HIV history data through a baseline questionnaire and review of participants’ medical records. All participants will undergo a screening ultrasound to confirm ultrasound eligibility (see exclusion criteria in the “Study participants” section).

Women will then undergo randomization into one of two study arms between 20 0/7 and 23 6/7 gestational weeks. Those participants who are screened during the window of randomization will be asked to return the next day for their randomization visit to allow them time to consider the risks and benefits of study participation and to discuss the study with their family. The trial will use a paper-based system of sequentially numbered, opaque, sealed envelopes to assign women 1:1 to active progesterone or placebo [30]. A statistician not otherwise associated with the study will design the scheme using random permuted blocks. All randomization documentation will be stored in a secure location. At the time of randomization, the research nurse will allocate the randomization envelopes in sequence to assign randomization identification numbers using standardized procedures. The statistician will compare the order of randomization to the original list order to verify strict order of randomization. Progesterone and placebo vaginal suppositories will be produced and individually packaged prior to being shipped to Zambia. Packaging will be non-descript and identical for the two products. Each progesterone lot and each placebo lot will have a unique identifying number produced from a random number generator that will be indexed in a secured database as either placebo or product, accessible only to the US-based compounding pharmacy and the unblinded statistician at interim analysis and end of study. Participants will be assigned randomization numbers that correspond to either an active or placebo lot number, to be dispensed by an on-site pharmacist masked as to treatment allocation. All other research staff members who have contact with participants will be masked to both treatment allocation and assigned product lot numbers. Participants will be instructed to begin daily self-administration of study product from the day of randomization (between 20^0^/_7_ and 23^6^/_7_ gestational weeks) until 36^6^/_7_ weeks, membrane rupture, or delivery, whichever is sooner. Women will be instructed on correct study product use at the randomization visit and at additional study visits, as needed. The on-site pharmacist will dispense suppositories with single-use applicators at 2-week intervals with an additional 14-day buffer to be replenished as needed at subsequent study visits. Each participant will receive an instructional sheet on correct product use and storage as well as a discreet carrier and plastic bags to facilitate return of used applicators at follow-up study visits. Participants will also be asked to complete a dose diary.

After randomization, participants will be asked to return to clinic every 2 weeks for monitoring of adherence by self-report, review of dose diaries, counting of unused product, and collection of used applicators. Trained technicians, masked to study participant and allocation, will test all returned applicators for evidence of vaginal insertion using a validated dye stain assay (DSA) [31] as detailed below. Standardized adherence counseling will be provided to all participants and tailored based on dose diary and self-reported low adherence.

### Qualitative activities

Women who decline enrollment into the randomized trial will be invited to participate in a one-time, face-to-face SSI to assess the following subject areas: (1) perceived risk of PTB; (2) health beliefs about and attitudes toward HIV and PTB, and toward a medication for the prevention of PTB; (3) acceptability of a daily vaginal medication compared to weekly intramuscular injections (the alternative formulation of antenatal progesterone); and (4) attitudes toward participation in a double-masked trial that uses a placebo control. We aim to sequentially interview at least 30 women who decline enrollment, with the goal of thematic saturation of data [32, 33].

Women who are enrolled and randomized to either progesterone or placebo, and who grant permission to be contacted separately about a qualitative study component, will be randomly selected to participate in three serial SSIs, held at enrollment, mid-study (28–32 weeks’ gestational age), and postpartum (prior to discharge). We aim to conduct interviews with women enrolled in the study to address the same domains outlined above, in addition to barriers and facilitators to: (1) adherence to study product; (2) returning used applicators, i.e., protocol adherence; and (3) retention in the study. If our random sample does not include women from the full range of adherence levels, we will apply purposeful sampling to capture women of underrepresented adherence levels for a single interview in their 3rd trimester. Our expected sample size for this group will be 30 women, based on expectations regarding saturation of qualitative themes [32, 33].

All interview sessions will be facilitated by a trained staff member with previous experience conducting qualitative interviews. Interviews will be held in a private room in the study clinic and are expected to last 30 min each. Semi-structured interview guides have been developed and will be used as an aid by the interviewer. Sessions will be audio-recorded and transcribed verbatim, with explicit approval provided by participants via informed consent. Where translations are needed, we will have independent reviewers review and translate those transcriptions to ensure accuracy.

All participants enrolled in this study will receive their usual antenatal and HIV care through the government healthcare system. ANC consists of an initial visit at less than 16 weeks gestation and follow-up visits no less frequently than at 24, 32, and 36 weeks gestation and delivery. GA will be determined by early ultrasound; cervical length measurements and fetal anatomic survey will be performed as early as possible before 24 weeks. During routine ANC visits, participants will receive the recommended care and treatment. At each study visit, study staff will screen participants for the presence of any adverse or serious adverse events. Medical care for any obstetrical complications encountered by trial participants will be provided at an appropriate Ministry of Health facility.

### Laboratory procedures

Trained laboratory technicians will test returned vaginal applicators using an inert dilute food dye (0.05% FD&C Blue No.1) that produces a distinctive streaked color when sprayed on polyethylene plastic applicators after vaginal insertion. Sensitivity and specificity of DSA for vaginal insertion is reported as 97 and 79%, respectively [31]. The DSA has been validated in sub-Saharan Africa, has been accurate at time points up to 4 months after insertion, and has shown no evidence of cross-contamination even when used and unused applicators were commingled in the same container [34].

### Primary outcome

Our primary outcome will be the proportion of women with adequate adherence to study product, defined as the return of at least 80% of provided vaginal applicators with DSA positivity.

### Sample size

We will pool participants from both arms for the primary adherence analysis and will also estimate adherence within each arm to investigate differences by treatment group. We used the normal approximation confidence limit approach to choose the sample size for this study, with the exact binomial confidence interval (CI) as a sensitivity approach. Using a 95% CI for the proportion of participants with adequate adherence and assuming an observed adherence rate of 80%, *n* = 140 provides a lower confidence limit above 70%. The target proportion of women achieving adequate adherence is 70–95%. Tables 1 and 2 presents the precision achieved for outcomes with an observed proportion of 50–95%.

**Table 1:** Anticipated precision with *n* = 140 (pooled)

	Observed proportion (adherence, retention)					
	0.5	0.6	0.7	0.8	0.9	0.95
Normal approximation of precision	±0.083	±0.081	±0.076	±0.066	±0.050	±0.036
Exact binomial 95% CI	0.41, 0.59	0.51, 0.68	0.62, 0.77	0.72, 0.86	0.84, 0.94	0.90, 0.98

**Table 2:** Anticipated precision with *n* = 70 (within arm)

	Observed proportion (adherence, retention)					
	0.5	0.6	0.7	0.8	0.9	0.95
Normal approximation of precision	±0.117	±0.115	±0.107	±0.094	±0.070	±0.051
Exact binomial 95% CI	0.38, 0.62	0.48, 0.72	0.58, 0.80	0.69, 0.89	0.80, 0.96	0.88, 0.99

### Statistical analysis plan

In addition to our primary adherence outcome, we will define uptake strictly as the proportion of women meeting initial (screening) eligibility criteria who are randomized in the trial; the sample used to estimate the probability of uptake will be larger than *n* = 140. If uptake in the pilot is very low, enrollment of a large trial might not be feasible. Low uptake might also threaten external validity and policy value. To quantify study retention, we will calculate the proportion of women randomized in the trial for whom we are able to ascertain the date of delivery and infant vital status at birth. For the present study, we will require a clinic visit to define retention success (either at delivery or postpartum). We will also explore whether this outcome can be reliably obtained through a home visit or even a telephone call by comparing reported data to medical records of those participants retained. Assuming the observed retention proportion is 95%, our sample size of 140 women will provide a 95% CI spanning 90–98% (Tables 1 and 2).

The proportion adherent will be compared between arms with an estimated risk ratio and corresponding 95% CI. We will also explore maternal demographic and health characteristics associated with adherence, uptake, and retention if there are sufficient numbers of cases. Factors found promising in an unadjusted log-binomial model will be candidates for a multivariable model. If cell counts are small, exact logistic regression will be utilized for sensitivity analysis. Secondary efficacy and safety outcomes will include: (a) delivery prior to 37 weeks gestation; (b) delivery prior to 34 weeks gestation; (c) birth weight <2500 g; (d) stillbirth; and (e) adverse events. In an exploratory analysis of these outcomes, we will conduct descriptive statistics of participant demographic and clinical features and of the proportion of participants experiencing the secondary outcomes of poor obstetrical and neonatal outcomes. Although our study is not powered for clinical efficacy, we will perform unadjusted analyses to determine whether a numerical difference in risk of these secondary efficacy and safety outcomes exists between women who were randomized to VP compared to those randomized to placebo. Exploratory analyses will be stated as such and presented as pilot effect estimates with a 95% CI; such analyses will provide useful results for planning a full-scale trial.

We will also investigate whether the use of a pre-randomization placebo run-in period [35] would be valuable in a full-scale efficacy trial. Several perinatal trials [36, 37] including multiple progesterone studies [18, 38, 39] have used a placebo run-in period to select participants who are most likely to be adherent prior to randomization. For a phase III VP trial, one could imagine a 2-week run-in period, where women are provided placebo/inert study product and only those returning applicators that met a certain adherence threshold would be randomized. While we do not intend to explicitly pilot this procedure, we will simulate it by dividing the adherence dataset into the first 2 weeks versus the remaining weeks and evaluate whether some initial threshold adherence (e.g., missing no more than one dose) is predictive of adequate adherence over the remaining study period. This exercise will allow us to evaluate the utility of a run-in in the overall context of phase III planning, when issues such as recruitment and representativeness are also being considered. Statistical approaches for diagnostic testing, such as receiver operating characteristic (ROC) curve analyses (plot of sensitivity versus 1-specificity), will be used to inform selection of the non-adherence cut-point. Sensitivity, specificity, positive predictive value, and negative predictive value of the chosen cut-point will be estimated with a corresponding 95% exact binomial CI.

### Qualitative analysis plan

Our primary qualitative objective is to identify common facilitators and barriers to trial uptake, vaginal product adherence, and retention in a clinical trial. We will first analyze data from women who decline enrollment independently from data among those who enroll. Then, findings from common baseline domains evaluated in both assessment groups will be compared to identify commonalities or discrepancies between decliners and enrollees. The longitudinal data assessed from interviews with enrolled participants will be compared across time and between those randomized to placebo versus progesterone. After translations and transcription, a team of coders with qualitative data analysis experience will use qualitative analysis software to organize the transcribed data. Transcript data will initially be organized based on the questions in the semi-structured guides. Coders will read and “memo” each transcript and compare memos to create a preliminary codebook. They will apply the codes to significant utterances, comparing their results periodically (such as every 4–5 interviews) to assess consistency between coders in order to refine a final codebook. They will examine codes and utterances for clusters of meaning to construct themes and maps reflecting categories and relationships among codes. They will then examine the themes identified from each dataset for contrasts and overlaps between them, which may lead to reconstructions of the existing themes or the emergence of new subthemes. A summary report of the analyzed SSI data will be created which will inform the development of the following materials for the planned full-scale efficacy and safety trial: study protocol, consent forms, adherence monitoring and counseling guide, and provider training materials.

### Ethical considerations

Participation in all study activities will be voluntary. Each participant will provide written, informed consent prior to enrollment. The study protocol has been approved by the University of Zambia Biomedical Research Ethics Committee and the University of North Carolina at Chapel Hill Institutional Review Board. Research staff who have contact with participants receive protection of human research participants training prior to participating in any study activities and every 2 years thereafter. Key research staff members complete Good Clinical Practice or Good Clinical Laboratory Practice training, as applicable.

It is expected that this study will expose participants to minimal risks. Self-administration of VP may cause mild discomfort to participants. Side effects and serious adverse events with VP administration are very rare. At each study visit, study staff will evaluate participants for social harms and adverse events. The severity of study-related adverse events and social harms will be graded using the National Institute of Health’s Division of AIDS Table for Grading the Severity of Adult and Pediatric Adverse Events. Information on adverse events or social harms that are related to the study drug and all serious adverse events will be documented on study data forms and routinely reported to the Principal Investigator or designee. If the PI, co-investigators, or their designees determine that study-related adverse events are occurring at an unexpected rate, they will assess the need for staff re-training, protocol amendment, or study cessation. All adverse events will be reported to the regulatory authorities listed above according to their individual guidelines. In the case of an emergency that would require unblinding, the statistician will disclose the allocation arm for the individual participant.

Participation in this study also raises the risks of loss of confidentiality and discomfort with the personal nature of questions, particularly when discussing HIV infection or sexual behaviors. At each step in the study, personnel will protect participant privacy and confidentiality to minimize these risks. All laboratory specimens, administrative forms, and study data will be identified by a random study number; data analysis will be performed using datasets linked to this number only.

Individual participants in the VP arm of the randomized trial may benefit from a reduction in PTB risk. All participants may benefit from enhanced health education and close clinical monitoring. Knowledge generated from this study has the potential to inform future clinical trials on the reduction of PTB risk among HIV-infected women, which may in turn enable policymakers worldwide to make informed decisions regarding effective interventions for the prevention of PTB.

## Discussion

The convergence of the HIV and preterm birth epidemics in sub-Saharan Africa has unveiled unanticipated consequences among women taking ART to prevent mother-to-child HIV transmission. As ART coverage continues to expand in the region, the burden of neonatal mortality and lifelong complications attributable to prematurity are likely to worsen. Global funding and research has succeeded in developing new technologies that improve outcomes for the preterm infant, yet many of these interventions are not applicable to resource-constrained settings, and advances in the area of prematurity prevention have been limited. Low-cost, high-impact interventions are desperately needed to prevent preterm birth among those at highest risk for prematurity and its consequences.

In preparation for a trial to test the effectiveness of vaginal progesterone for the prevention of preterm birth among HIV-infected women, this pilot study will allow us to estimate key parameters of trial feasibility: uptake, adherence, and retention. It will also provide insight into rates of adverse events and perhaps preliminary efficacy. Finally, it will help us develop and test study procedures. The potential knowledge gained from this research could influence health policy and potentially prevent thousands of preterm births per year in Zambia alone. If prenatal progesterone is found to reduce the risk of preterm delivery faced by the 1.5 million HIV-infected women who become pregnant each year worldwide, elimination of infant HIV infection might be achieved without paying the price in prematurity-related death and disability.

## Abbreviations

ANC: Antenatal care
ART: Antiretroviral therapy
CI: Confidence interval
DSA: Dye stain assay
GA: Gestational age
HIV: Human immunodeficiency virus
PrEP: Pre-exposure HIV prophylaxis
PTB: Preterm birth
RCT: Randomized controlled trial
SSI: Semi-structured interview
UTH: University Teaching Hospital
VP: Vaginal progesterone
